# Feasibility of using the UDS v3.0 T‐cog longitudinally to identify perioperative neurocognitive disorders in older adults

**DOI:** 10.1002/alz70858_098241

**Published:** 2025-12-24

**Authors:** Rachel R Wu, Mika Rockholt, Christina L Bi, Elaine Zhu, Hamleini Martinez, Ekow B Commeh, Romario B Denoon, Gabrielle Bruno, Braden V Saba, Daniel Waren, Raven Perez, Wonyoung Park, Courtney O'Brien, Vinay K Aggarwal, Joshua C Rozell, Karyn Marsh, David Furgiuele, William Macaulay, Ran Schwarzkopf, Evan T Schulze, Arjun V. Masurkar, Alok Vedvyas, Allal Boutajangout, Thomas Wisniewski, Ricardo S. Osorio, Lisa V Doan, Jing Wang

**Affiliations:** ^1^ NYU Grossman School of Medicine, New York, NY, USA; ^2^ NYU Langone Health, New York, NY, USA; ^3^ NYU Langone Medical Center, New York, NY, USA; ^4^ New York University Grossman School of Medicine, New York, NY, USA; ^5^ Alzheimer's Disease Research Center, New York University Langone Health, New York, NY, USA; ^6^ Center for Cognitive Neurology, New York University Langone Health, New York, NY, USA; ^7^ NYU Langone School of Medicine/ Alzheimer's Disease Research Center, New York, NY, USA

## Abstract

**Background:**

Perioperative neurocognitive disorder (NCD), or measurable functional cognitive decline is overrepresented in patients 65 years of age, and older. The risk factors for NCD development are still not fully characterized making it important to measure cognitive function longitudinally and comprehensively to better understand which patients develop NCD and the associated risk factors. Other studies investigating NCD using virtual assessments have used brief cognitive screeners, including the T‐MOCA, MMSE, and Modified Telephone Interview for Cognitive Status (TICS‐M) that may lack sensitivity. This ongoing prospective observational study is the first to use the National Alzheimer's Coordinating Center (NACC) UDS v3.0 T‐cog exam administered virtually to test the feasibility of capturing a robust cognitive assessment up to 3 months post‐operation.

**Method:**

Patients aged 65 years and older who were scheduled for hip or knee arthroplasty took the assessment via phone or video Zoom call with trained research staff to establish a baseline cognitive function, before surgery. Follow‐up assessments were administered 1 week, 1 month, and 3 months post‐operatively. NCD was defined as a change in ≥ –1 SD in Z‐scores in at least two cognitive domains.

**Result:**

91 patients, with a mean age of 73.2 years, were analyzed in our study. The average number of years of education was 17.4 and 59.3% were female. 78.0% of subjects completed their 1‐week post‐operative cognitive assessment, 83.0% completed the 1‐month post‐operative assessment, and 84.5% completed the 3‐month post‐operative assessment. Rates of NCD at 1 week, 1 month, and 3 months post‐operatively were 31.9%, 29.9%, and 21.7%, respectively.

**Conclusion:**

The UDS v3.0 T‐cog has a high completion rate up to 3 months following surgery. It is a well validated exam for assessing patients’ cognitive abilities and represents a more comprehensive exam compared to current assessments used in this setting, it may be more sensitive to cognitive changes. Future work includes comparing UDS v3.0 T‐cog sensitivity against existing cognitive assessments used in the perioperative setting.

